# CCAAT/enhancer-binding protein delta regulates miRs-4257 and 3156 to attenuate the interleukin 12 through small extracellular vesicle transmission in glioblastoma

**DOI:** 10.1186/s12935-026-04225-2

**Published:** 2026-02-25

**Authors:** Yu-Yi Chu, Chiung-Yuan Ko, Shao-Ming Wang, Wei-Jan Wang, Chih-Yang Wang, Feng-Wei Chen, Hsin-Yin Liang, Ju-Ming Wang

**Affiliations:** 1 Department of Biotechnology and Bioindustry Sciences, College of Bioscience and Biotechnology, National Cheng Kung University, Tainan, 701 Taiwan; 2 School of Medicine, College of Medicine, National Sun Yat-Sen University, Kaohsiung, 80424 Taiwan; 3 Institute of Biomedical Sciences, College of Medicine, National Sun Yat-Sen University, Kaohsiung, 80424 Taiwan; 4 Department of Biomedical Science and Environment Biology, College of Life Science, Kaohsiung Medical University, Kaohsiung, 80424 Taiwan; 5 Graduate Institute of Biomedical Sciences, China Medical University, Taichung, 404333 Taiwan; 6 Neuroscience and Brain Disease Center, China Medical University, Taichung, 404333 Taiwan; 7 Department of Biological Science and Technology, China Medical University, Taichung, 40676 Taiwan; 8 Research Center for Cancer Biology, China Medical University, Taichung, 40676 Taiwan; 9 Program for Cancer Molecular Biology and Drug Discovery, College of Medical Science and Technology, Taipei Medical University, Taipei, Taiwan; 10 Graduate Institute of Medical Sciences, College of Medicine, Taipei Medical University, Taipei, Taiwan; 11 Graduate Institute of Medicine, College of Medicine, Kaohsiung Medical University, Kaohsiung, Taiwan

**Keywords:** Glioblastoma multiforme, MicroRNA, CEBPD, SEV transmission, IL-12

## Abstract

Glioblastoma multiforme (GBM) is a heterogeneous and malignant brain tumor characterized by an immunosuppressive microenvironment, notably with diminished M1 macrophage activity. MicroRNAs serve as post-transcriptional regulators and have been implicated in influencing tumor progression. However, the interaction between microRNAs and tumor-associated macrophages in glioma remains less characterized. CCAAT/enhancer-binding protein delta (CEBPD) could act as an oncogenic factor in GBM and regulate microRNA transcription, thereby impacting tumorigenesis. In this study, we demonstrated that glioma CEBPD directly binds to and activates the promoter regions of miR-4257 and miR-3156, located within the genes *ADAMTSL4* and *ANKRD30BP3*, respectively. These microRNAs are transmitted via small extracellular vesicles (sEVs) and target macrophages, specifically binding to the 3’-untranslated regions (UTRs) of interleukin 12 (IL-12) *p35* and *p40* mRNAs, thereby reducing IL-12 transcription and expression in macrophages. Furthermore, our results show that sEV antisense miR-4257 and miR-3156 diminish the M1 macrophage phenotype. In animal models, co-inoculation of glioma cells with antisense miR-4257 and miR-3156 or CEBPD knockdown, along with M1 macrophages, leads to reduced tumor growth and enhanced M1 macrophage activation. These findings suggest that glioma CEBPD can contribute to immunosuppression by regulating miR-4257 and miR-3156, which target IL-12 in macrophages through sEV transmission. This research offers new insights into the relationship between glioma and immunosuppression, highlighting potential therapeutic avenues for enhancing anti-tumor immunity in GBM.

**Supplementary Information:**

The online version contains supplementary material available at 10.1186/s12935-026-04225-2.

## Introduction

Glioblastoma multiforme (GBM) is a highly heterogeneous and aggressive form of brain cancer [1, 2] with no effective treatment and a median survival of only 18–21 months [3]. This poor prognosis is attributed to immunosuppression and drug resistance within the tumor microenvironment [4, 5]. Surgery is a common clinical treatment for glioma, especially glioblastoma, and postoperative radiotherapy and chemotherapy can improve patient outcomes. Tumor-associated macrophages (TAMs) are categorized as glioblastoma-suppressive M1-like and glioblastoma-supportive M2-like polarized cells. Several therapeutic strategies focus on reversing or reprogramming the population of M2-like TAMs into M1-like TAMs in GBM [6, 7], emphasizing the importance of macrophage polarization in effective GBM treatment.

Several types of non-coding RNAs (ncRNAs), including microRNAs (miRNAs), long non-coding RNAs (lncRNAs), and circular RNAs (circRNAs), have been identified and play critical roles in cell growth, proliferation, and metabolism through multiple mechanisms [8–10]. MiRNAs are 20–25 nucleotide-long ncRNAs that regulate gene expression by binding to the 3’-untranslated region (3’-UTR) of target mRNAs, leading to gene silencing at the post-transcriptional level. They are involved in various cellular processes in normal physiology and disease, particularly in tumor progression [11, 12]. Extracellular vehicles (EVs), including exosomes, small extracellular vesicles (sEVs), microvesicles, and apoptotic bodies, are lipid-based vesicles [13]. Among these, exosomes and other sEVs are nano-sized [14] and have been reported to carry miRNAs, RNAs, and proteins that influence tumor progression [14]. In cancer, extensive studies have demonstrated a dynamic communication cycle between tumor cells and immune cells. Tumor cells can induce tumor-associated macrophage (TAM) polarization, and in turn, polarized TAMs enhance tumor malignancy. In this crosstalk, tumor-derived sEV ncRNAs regulate immune responses, including macrophage polarization and suppression of T and natural killer cell activity. On the contrary, TAM-derived sEV ncRNAs contribute to proliferation, metastasis, chemoresistance of cancer cells, and immunosuppression.

CCAAT/enhancer-binding protein delta (CEBPD) functions as either a pro-tumor role in glioblastoma [2, 15], urothelial carcinoma [16–18] and gallbladder cancer [19], or anti-tumor role in hepatocellular carcinoma [20, 21], cervical carcinoma [22, 23], prostate carcinoma [24], leukemia [25] and breast tumors [26]. Its biological functions involve regulating inflammation [26, 27], anti-apoptosis [27], oxidative stress production [28, 29], and migration [28]. Additionally, CEBPD is implicated in the progression of neurological diseases [28–30], cardiovascular diseases [31], and rheumatoid arthritis [32]. However, regarding serving a pro-tumor role, it remains unclear whether CEBPD can influence glioma progression through immunosuppression, particularly by modulating TAM function. Previously, CEBPD was shown to regulate microRNA expression, influencing the expression of neurotrophic factor and angiogenesis-related genes in astrocytes [15, 33]. Additionally, CEBPD-induced miRNAs have been found to mediate epigenetic regulation in leukemia, leading to cell cycle arrest and cell death [25]. However, the role of CEBPD-regulated miRNAs in glioma cells and their potential involvement in mediating immunosuppression remains to be investigated.

Here, our results demonstrated that glioma CEBPD regulates miR-4257 and miR-3156, which are secreted to TAMs via sEV transmission, where they bind to the 3’-UTR of IL-12 *p35* and *p40* mRNA, leading to the decrease of antitumor IL-12 expression in TAM. Furthermore, we showed that antisense oligonucleotides targeting miR-4257 and miR-3156 can increase the population of M1 macrophages and reduce tumor volume. These findings suggest that targeting sEV miR-4257 and miR-3156 effectively alleviates immunosuppression and inhibits cancer progression by enhancing IL-12 expression.

## Materials and methods

### Cell culture

The human glioblastoma cell lines U87MG and U373MG were obtained from the American Type Culture Collection (ATCC, Manassas, VA, USA). The cells were cultured in Dulbecco’s Modified Eagle Medium (DMEM; GIBCO, Waltham, MA, USA) supplemented with 10% fetal bovine serum (FBS; CORNING, REF 35–010-CV, Glendale, AZ, USA) and 1% penicillin–streptomycin (GIBCO, Waltham, MA, USA). THP-1 cells were cultured in RPMI 1640 medium supplemented with 5% fetal bovine serum (FBS), streptomycin (100 µg/ml), and penicillin (100 U/ml). To differentiate THP-1 cells into THP-1–derived macrophages, 1 × 10^6^ cells were seeded into a six-well plate and treated with 320 nM PMA (Sigma) for 24 h.

### Animal model

Female NOD (non-obese diabetic)/severe combined immunodeficient (SCID) mice aged 6 to 8 weeks were purchased from the Laboratory Animal Center at National Cheng Kung University. U87MG cells (5 × 10^6^) carrying shLacZ, shCD, antisense miR-3156, or antisense miR-4257 IPTG-inducible vectors were mixed with M1-like bone marrow-derived macrophages (5 × 10^5^) in 100 μL of PBS and subcutaneously inoculated into the right flanks of mice. On day 14, tumor size was measured using external calipers, and tumor volume was calculated using the standard formula: V = (w × l^2^) × 0.52, where l is the length and w is the width of the tumor. Four to six animals were used per group for tumor volume measurements. The raw data are provided in the Supplementary Data.

### miRNA PCR

RNA extraction was carried out using TRIzol, followed by cDNA synthesis using the TaqMan MicroRNA Reverse Transcription Kit. The resulting cDNA was analyzed through real-time PCR with the TaqMan MicroRNA Assay Kit (Applied Biosystems, Waltham, MA, USA). Quantitative PCR (qPCR) was subsequently performed using the CFX96 Touch Real-Time PCR Detection System (Bio-Rad).

### Cloning and constructs

A detailed description of all constructs and vectors used in our study has now been included in the Materials and Methods section as follows: Fragments for expressing miRs and AsmiRs were generated by annealing synthesized oligonucleotide pairs as follows. For miR-3156, the oligonucleotides were 5’ -CCGGTGTCTCCCACTTCCAGATCTTTGGATCCAAAGATCTGGAAGTGGGAGACATTTTT-3’ and 5’-AATTAAAAATGTCTCCCACTTCCAGATCTTTGGATCCAAAGATCTGGAAGTGGGAGACA-3’. For miR-4257, the sequences were 5’-CCGGCTCAGTCCCCACCTCTGGGGATCCCCAGAGGTGGGGACTGAGTTTTT-3’ and 5’-AATTAAAAACTCAGTCCCCACCTCTGGGGATCCCCAGAGGTGGGGACTGAG-3’. The oligonucleotide pair used to generate AsmiR-3156 was 5’-CCGGAAAGATCTGGAAGTGGGAGACAGGATCCTGTCTCCCACTTCCAGATCTTTTTTTT-3’ and 5’-AATTAAAAAAAAGATCTGGAAGTGGGAGACAGGATCCTGTCTCCCACTTCCAGATCTTT-3’. AsmiR-4257 was produced using 5’-CCGGCCAGAGGTGGGGACTGAGGGATCCCTCAGTCCCCACCTCTGGTTTTT-3’ and 5’-AATTAAAAACCAGAGGTGGGGACTGAGGGATCCCTCAGTCCCCACCTCTGG-3’. The annealed fragments were then ligated into the lentiviral expression vector pLAS1w.3xLacO. The lentiviral expression vector pLAS1w.3xLacO was obtained from the National RNAi Core Facility at the Genomic Research Center, Institute of Molecular Biology, Academia Sinica (Taiwan). The 3’UTRs of IL-12 p35 and IL-12 p40 were amplified using primers engineered to include restriction sites for downstream cloning. The IL-12 p35 3’UTR was amplified using an XbaI-containing forward primer (5’-TCTAGAAAAGCGAGGTCCCTCC-3’) and an EcoRV-containing reverse primer (5’-GATATCGATGCTTTCATGATTACCAAG-3’). The IL-12 p40 3’UTR was amplified with a forward primer containing an XbaI site (5’-TCTAGAGTTCTGATCCAGGATGAAA-3’) and a reverse primer containing an EcoRV site (5’-GATATCGATTACAAAGAAGAGTTTTTATTAGTT-3’). PCR products were subsequently cloned into the pGL3 luciferase reporter vector to assess regulatory activity. Mutant reporter constructs were generated using the QuikChange Site-Directed Mutagenesis Kit (Stratagene, CA, USA). Mutagenesis of the IL-12 p35 3’UTR was performed using the primer pair 5’-TACATCCACATGATATGTAGAATCAAGTATTTTTG-3’ and 5’-CAAAAATACTTGATTCTACATATCATGTGGATGTA-3’. Mutations within the IL-12 p40 3’UTR were introduced using primers 5’-TCTGGAAGGCAGAACGATATCAAGATTCAAGAGAG-3’ and 5’-CTCTCTTGAATCTTGATATCGTTCTGCCTTCCAGA-3’.

### ChIP assay

In brief, knockdown cells were fixed with 1% formaldehyde for 15 min to induce crosslinking. The chromatin was then extracted and sonicated to generate DNA fragments averaging less than 1000 bp. Immunoprecipitation of the DNA fragments was performed using antibodies against CEBPD and control immunoglobulin G, with incubation at 4 °C for 16 h. Following the reversal of the crosslinking, the immunoprecipitated chromatin was amplified using primers targeting specific regions of the genomic loci of the target genes.

### Immunofluorescence assay

Fixed brain tissues were embedded in OCT compound and sectioned. The OCT was dissolved in PBS for 10 min, and the brain sections were subjected to antigen retrieval at 80 °C for 15 min. The sections were then blocked for 1 h at room temperature using a blocking buffer containing 10% normal donkey serum in PBS with 0.1% Tween 20. After blocking, the sections were incubated with specific primary antibodies at 4 °C for 16–20 h. The following day, the sections were washed three times with TBST for 10 min each, then incubated with Alexa Fluor 488- or 555-conjugated secondary antibodies in PBS for 1 h. After three additional 10-min washes with TBST, the sections were mounted with ProLong Gold Antifade Reagent, with or without DAPI, and left at 23 °C for 10 min.

### Luciferase reporter assay

Cells were transfected using the TurboFect Transfection Reagent (Thermo Scientific, Pittsburgh, PA, USA) according to the manufacturer’s instructions. The total amount of DNA for each experiment was adjusted to match that of the respective backbone vector. After transfection, cell lysates were collected and processed using the luciferase assay system following the manufacturer’s instructions (Promega, Madison, WI, USA). For the assay, 50 µL of cell lysate, 50 µL of luciferin, and 180 µL of luciferase assay reagent were combined in a luminometer tube. After vertexing, the tube was placed in the luminometer, and luciferase activity was measured.

### Small macrovesicle preparation

Cell culture media will be centrifuged at 18,000 g for 10 min to remove cellular debris. The supernatant will then be collected and centrifuged at 18,000 g for 45 min at 15 °C. The resulting sEV pellet will be resuspended in 500 µL of phosphate-buffered saline, centrifuged again at 18,000 g for 45 min, and snap-frozen in liquid nitrogen. Multiple sEV preparations will be pooled for RNA and protein extraction.

### Quantitative PCR (qPCR)

Total RNA was extracted using TRIsure, and cDNA was synthesized via reverse transcription with SuperScript III. The cDNA was then mixed with oligonucleotide primers and enzymes, and qPCR was performed using the CFX96 Touch Real-Time PCR Detection System (Bio-Rad). The results were analyzed during the qPCR process.

### Western blot

The treated cells were collected and lysed using a modified RIPA buffer containing 50 mM Tris–HCl (pH 7.4), 150 mM NaCl, 1 mM EDTA, 1% NP-40, 0.25% sodium deoxycholate, 1 mM DTT, and a protease inhibitor cocktail. Protein extracts were resolved by SDS-PAGE on an 8–12% polyacrylamide gel, transferred onto a PVDF membrane, and incubated overnight at 4 °C with primary antibodies specific to the target proteins. The membrane was then incubated with HRP-conjugated secondary antibodies, and the protein signals were detected using the Enhanced Chemiluminescence Western Blot System (Thermo Scientific, Rockford, IL, USA).

### Survival analysis

Survival information for the TCGA Glioblastoma Multiforme (GBM) cohort was obtained from the Genetic Determinants of Cancer Patient Outcome database (https://tcga-survival.com/; CSV file downloaded). GBM patients were divided into CEBPD high and low groups by the best cutoff value. Survival curves were generated using GraphPad Prism 8.0, and statistical significance was calculated by a two‐sided log-rank test.

### Gene expression analysis

Normalized mRNA expression data (RNA-Seq, RSEM normalized counts) for the TCGA GBM cohort were downloaded from the FireBrowse database (http://firebrowse.org/; “illuminahiseq_rnaseqv2-RSEM_genes_normalized” file, MD5 verified). Samples were divided into CEBPD high and low groups by the median cutoff value. *IL12A* mRNA expression levels were plotted using GraphPad Prism 8.0, and statistical significance was determined by a two-tailed unpaired Student’s t test. Correlation analyses of gene expression were performed using GraphPad Prism 8.0, and P values were calculated using the nonparametric Spearman correlation test.

### MicroRNA expression analysis

Cell-free plasma microRNA expression profiles from healthy individuals (n = 73) and GBM patients (n = 45) were obtained from the GSE184472 dataset in the Gene Expression Omnibus (GEO) database. Expression levels of hsa-miR-4257 and hsa-miR-3156 were plotted using GraphPad Prism 8.0, and statistical significance was calculated using a two-tailed unpaired Student’s t test.

### In situ hybridization

Non-radioactive in situ hybridization was performed on 10 µm sections of mouse brain tissue using a digoxigenin-labeled, locked nucleic acid-modified detection probe and the IsHyb in situ hybridization kit (BioChain, Hayward, CA), following the protocol provided by Exiqon. Briefly, the sections were deparaffinized, hydrated, treated with proteinase K (10 µg/ml; Qiagen) at 37℃ for 15 min, and fixed with 4% paraformaldehyde for 20 min. Pre-hybridization was performed by incubating the sections in a pre-hybridization solution at 50℃ for 3 h, followed by hybridization with a digoxigenin-labeled, locked nucleic acid-modified detection probe at 50℃ for 16 h. After hybridization, the sections were incubated with a PBS-diluted anti-digoxigenin-AP antibody at room temperature for 1 h. This was followed by sequential washes in 2X standard saline citrate (SSC), 1.5X SSC, and 0.2X SSC. The sections were then incubated in a blocking solution for 1 h at room temperature, washed three times with PBS, and subsequently washed twice with 1X alkaline phosphatase buffer. Visualization of the hybridization signal was achieved using the NBT/BCIP solution.

### CCK8 assay

Cells were seeded in 96-well plates and incubated for 24 h. U87MG cells were then treated with or without As3156 or As4257 for an additional 24 h. Subsequently, a tenfold dilution of the CCK-8 reagent in the culture medium was added to the cells and incubated at 37 °C in a 5% CO₂ incubator for 1 h. The optical density (OD) was measured at 450 nm using an ELISA reader.

### Statistical analysis

Data were collected from three independent cell culture experiments and from at least four to six mice for animal studies. Statistical analyses were performed using Prism software (version 6). Results from replicate experiments are presented as the mean ± SD. An unpaired Student’s t-test was used to assess statistical significance, with the following thresholds: *p < 0.05, **p < 0.01, and ***p < 0.001.

## Results

### CEBPD in glioblastoma inactivates M1-like macrophages, thereby promoting tumor progression in vivo

**Fig. 1:**
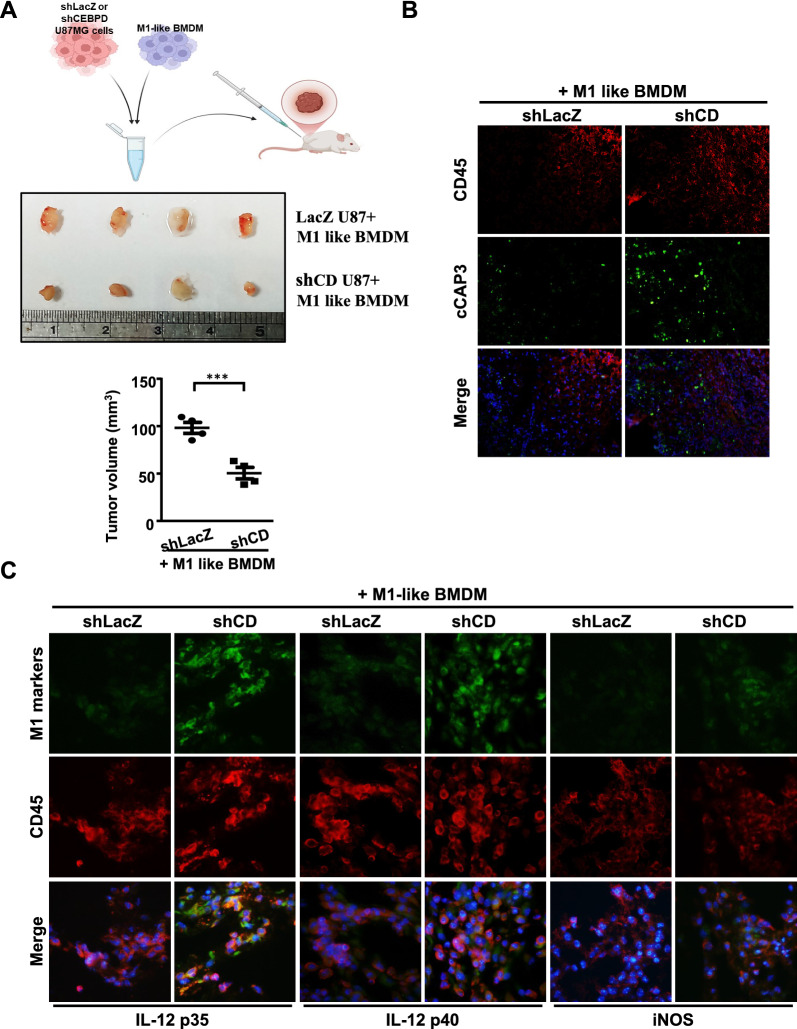
Inhibiting CEBPD in U87MG cells activates M1-like macrophage and promotes its anti-tumor activity in vivo. blocking CEBPD in U87MG cells reduced tumor volume in vivo. U87MG cells bearing IPTG-inducible shLacZ (LacZ-U87MG) or shCEBPD (CD-U87MG) expression vectors were cultured in IPTG-containing medium for 96 h. These cells were then subcutaneously co-inoculated with M1-like bone marrow-derived macrophages into NOD-SCID mice. (**A**) schematic illustration of the animal model experiment for glioma demonstrating that CEBPD inactivation suppresses M1-like macrophage activation (Upper panel). On day 14, tumor size was measured with external calipers, and tumor volume was calculated using the standard formula: V = (w × l^2^) × 0.52, where l is the length and w is the width of the tumor (n = 4 per group). Quantitative data are presented as means ± SD; ***p < 0.001. (**B**) histological analysis of tumor cell apoptosis in xenograft shLacZ- or shCEBPD-U87MG cells co-inoculated with M1-like macrophages was performed. Tumor tissues were subjected to immunofluorescence staining with antibodies specific for cleaved caspase 3 (cCap3; active caspase 3) and CD45 (a marker for xenograft M1 macrophages). DAPI staining was used to visualize cell nuclei. (**C**) immunofluorescent staining of M1 hallmark effector molecules, IL-12 p35, p40, and iNOS, was performed on M1 macrophages. Frozen sections of xenografts containing M1 macrophages and U87MG cells bearing IPTG-inducible shLacZ (LacZ-U87MG) or shCEBPD (CD-U87MG) expression vectors were immunostained with antibodies against CD45 (a marker for xenograft M1 macrophages), IL-12 p35, p40, and iNOS. DAPI staining was used as a nuclear counterstain

Our previous results demonstrated that CEBPD contributes to anti-apoptotic and pro-angiogenic effects in glioblastoma cells [15, 27]. In this study, we further observed that the levels of *CEBPD* transcripts were significantly higher in grade III/IV GBM patients compared to normal brain lysates (Supplementary Fig. 1). These findings suggest that CEBPD may exert a potential tumorigenic effect in glioblastoma. Interestingly, tumor-associated macrophages (TAMs) in lower-grade astrocytomas were strongly stained with the M1 marker [34]. However, it remains unclear whether CEBPD in glioma can suppress the antitumor immunity of M1-like macrophages in GBM. To investigate the role of CEBPD in GBM malignancy, we employed a loss-of-function approach to reduce CEBPD expression in human GBM cells. Interestingly, we found that the xenograft tumor volume of CEBPD-deficient cells co-inoculated with M1-like macrophages was significantly reduced compared to the control group (Fig. 1A). Moreover, significant caspase-3 activity was detected in the tumor regions containing CEBPD-deficient cells (Fig. 1B).

To further investigate whether CEBPD inhibits the antitumor activity of M1-like macrophages by inactivating the M1 phenotype in GBM, we examined key hallmark genes of M1-like polarized macrophages in tumor histology. Interleukin 12 (IL-12p70 or IL-12), a heterodimeric consisting of a heavy (p40) and a light (p35) chain subunit, is a pro-inflammatory and antitumor cytokine [35–37]. Higher levels of IL-12 p35, IL-12 p40, and iNOS were detected in the macrophages co-inoculated with CEBPD-deficient glioma cells in vivo (Fig. 1C). These results suggest that CEBPD in GBM promotes tumor progression by reducing the properties of M1-like macrophages.

### miR-4257 and miR-3156 are CEBPD-responsive miRNAs that repress IL-12 expression

**Fig. 2:**
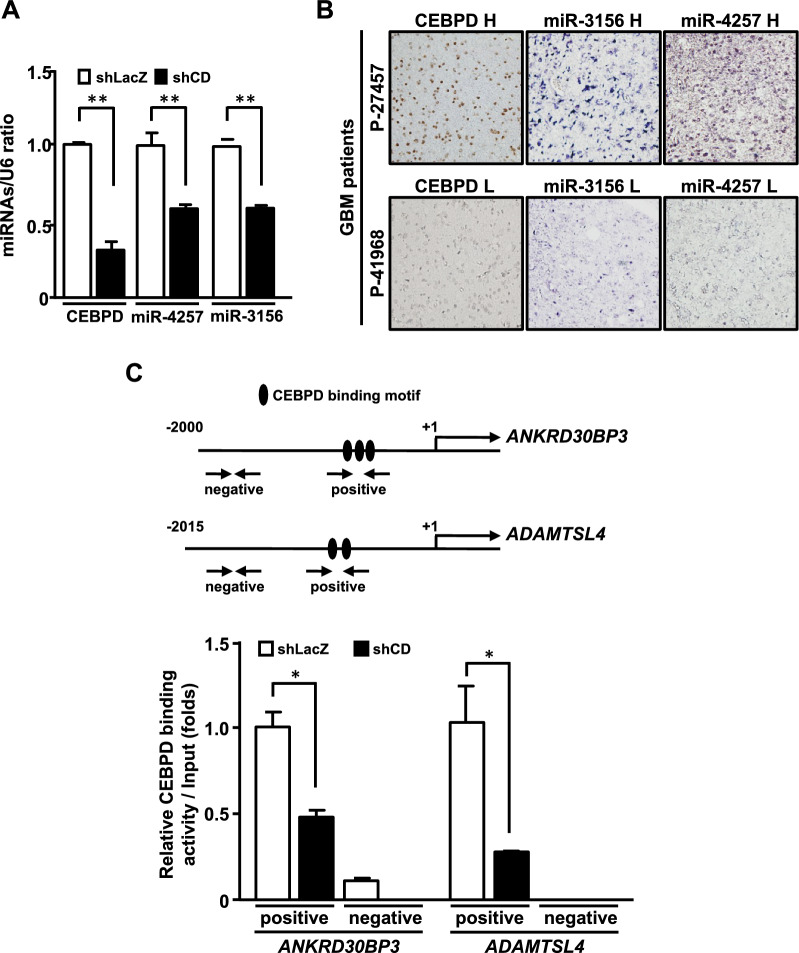
CEBPD activates the transcription of miR-4257 and miR-3156 in glioblastoma. (**A**) loss of CEBPD represses the expression of miR-4257 and miR-3156 in U87MG cells. After 96 h of IPTG treatment, total RNA was harvested from U87MG cells harboring IPTG-inducible shLacZ (LacZ) or shCEBPD (CD) expression vectors. the levels of miR-4257 and miR-3156 were analyzed by qPCR. (**B**) A positive correlation between CEBPD and miR-4257/miR-3156 expression is observed in GBM patients. immunohistochemical staining for CEBPD and in situ hybridization staining for miR-4257 and miR-3156 were performed on three consecutive tissue slides from GBM patients. Representative staining images revealed that a patient (P-27457) with higher levels of CEBPD (CEBPD H) exhibited abundant miR-4257 (miR-4257 H) and miR-3156 (miR-3156 H). Conversely, a patient (P-41968) with lower levels of CEBPD (CEBPD L) displayed reduced levels of miR-4257 (miR-4257 L) and miR-3156 (miR-3156 L). (**C**) CEBPD directly binds to the ANKRD30BP3 and ADAMTSL4 promoters in vivo. A ChIP assay was conducted using U87MG cells with IPTG-inducible shLacZ (LacZ) or shCEBPD (CD) expression vectors. After 96 h of IPTG treatment, chromatin from these cells was immunoprecipitated using a specific antibody against CEBPD. A schematic diagram illustrates the locations of primers used to detect the ANKRD30BP3 and ADAMTSL4 promoters by PCR. The precipitated DNA was amplified by PCR using primers targeting the ANKRD30BP3 and ADAMTSL4 promoter regions containing putative CEBPD-binding motifs (positive primers) and a negative control primer. The data represent the mean ± standard error of three independent experiments, each performed in triplicate. (*p < 0.05 and **p < 0.01, Student’s t-test)

Next, we aimed to dissect how CEBPD leads to the inactivation of M1 macrophages in GBM. Since miRNAs could play a key role in regulating cell–cell communication, the microarray profiles of reduction of CEBPD expression in U373MG cells were conducted and applied to be further analyzed (Supplementary Fig. 2). We identified 26 miRNAs that were upregulated by CEBPD in U373MG cells but not in THP-1 cells (Supplementary Table 1). The GEO dataset of glioma revealed that the levels of miR-4257 and miR-3156 were higher in GBM tissues compared to normal brain tissues (Supplementary Fig. 3). We further detected significantly higher levels of these two miRNAs in U87MG cells compared to human peripheral blood monocytes (Supplementary Fig. 4). Using the TargetScan prediction program, miR-4257 and miR-3156 were predicted to target the 3’-UTR of IL-12 *p35* and *p40* mRNA. Moreover, attenuating CEBPD reduced the expression of miR-4257 and miR-3156 in U87MG cells (Fig. 2A). Immunohistochemistry and in situ hybridization also indicated that GBM patients with abundant expression of CEBPD exhibited higher levels of miR-4257 and miR-3156 (Fig. 2B). These findings suggested a positive correlation between CEBPD and these two miRNAs in GBM. We next examined whether CEBPD can regulate miR-4257 and miR-3156 by directly binding to their promoters. Intragenic miR-4257 and miR-3156 are located within the genes *ADAMTSL4* and *ANKRD30BP3*, respectively. An in vivo DNA binding assay showed that the loss of CEBPD attenuates its binding activity to the promoter regions of the genes encoding miR-4257 and miR-3156 (Fig. 2C). Collectively, CEBPD activated the transcription of miR-4257 and miR-3156 in GBM.

### miR-4257 and miR-3156 directly target the IL-12 p35 and p40 3’-UTRs to suppress IL-12 expression

**Fig. 3:**
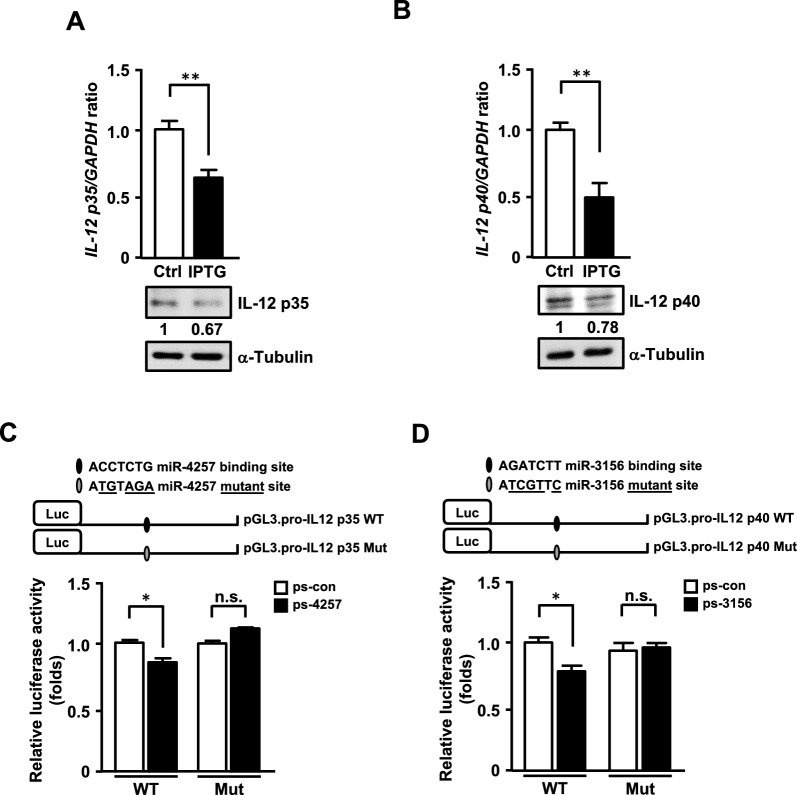
miR-4257 and miR-3156 suppress IL-12 expression by targeting its 3’- UTR region. (**A**) miR-4257 and (**B**) miR-3156 attenuate the expression of IL-12 p35 and p40, respectively. qRT-PCR and western blot analyses confirmed that the mRNA and protein levels of IL-12 p35 and p40 were reduced in BV2 cells with induced expression of miR-4257 and miR-3156, respectively. (**C**) A region of the IL-12 p35 3’-UTR is predicted to be a target of miR-4257. The seed regions are indicated by the box (upper panel). Luciferase activity of reporter constructs was measured after co-transfection with control (ps-con) or miR-4257 (ps-4257) expression vectors. (**D**) A region of the IL-12 p40 3’-UTR is predicted to be a target of miR-3156. The seed region is indicated by the box (upper panel). Luciferase activity of reporter constructs was measured after co-transfection with pre-miR-3156. the data are presented as the mean ± standard error of three independent experiments, each performed in triplicate. (*p < 0.05 and **p < 0.01, Student’s t-test)

miR-4257 was predicted to target the 3’ UTR of IL-12 *p35* mRNA, while miR-3156 was predicted to target the 3’ UTR of IL-12 *p40* mRNA. To verify that miR-4257 and miR-3156 can indeed repress IL-12 expression, IPTG-inducible miR-4257 and miR-3156 systems were established. The mRNA and protein levels of IL-12 p35 and IL-12 p40 were reduced upon induction of miR-4257 or miR-3156, respectively (Fig. 3A and 3B). A miR-4257 binding motif was identified in the 3’-UTR of IL-12 *p35* mRNA (Fig. 3C, top panel). Meanwhile, a miR-3156 binding motif was found in the 3’-UTR of IL-12 *p40* mRNA (Fig. 3D, top panel). To confirm that the 3’-UTRs of IL-12 p35 and p40 mRNAs are indeed targeted by miR-4257 and miR-3156, respectively, we constructed luciferase reporter plasmids containing either the wild-type or mutated seed sequences of the IL-12 *p35* or *p40* 3’- UTR. The reporter assays were conducted by co-transfecting IL-12 p35 or p40 3’-UTR reporters with miR-4257 or miR-3156 expression vectors in THP-1 cells. We found that miR-4257 repressed the activity of the wild-type IL-12 p35 3’-UTR reporter, but these effects were lost in constructs with individual mutations in the miR-4257 binding motifs (Fig. 3C). Similar regulation was observed by co-transfecting IL-12 p40 3’-UTR reporters with the miR-3156 expression vector in THP-1 cells (Fig. 3D). Taken together, these results demonstrate that miR-4257 and miR-3156, activated by CEBPD, can target the IL-12 *p35* and *p40* 3’-UTR and suppress their expression.

### CEBPD-responsive miR-4257 and miR-3156 in GBM regulate M1 macrophage effector molecules in vitro

**Fig. 4:**
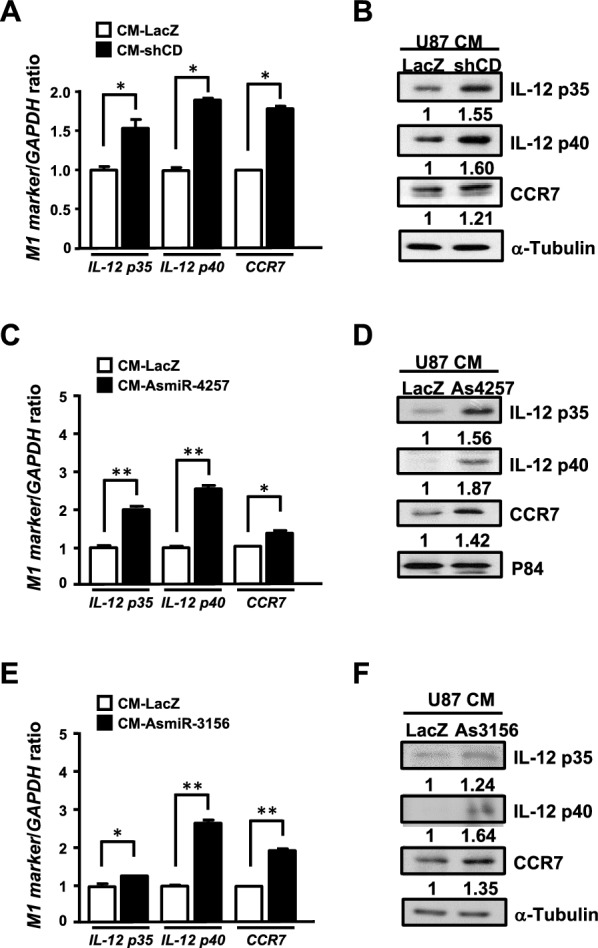
Blocking CEBPD, miR-4257, and miR-3156 in U87MG cells increases the expression of M1 macrophage markers in vitro. Conditioned medium (CM) was harvested from U87MG cells with IPTG-inducible shLacZ (LacZ), shCEBPD (CD), antisense miR-4257 (As4257), or antisense miR-3156 (As3156) expression vectors after IPTG treatment for 96 h. Total RNA and protein were harvested from bone marrow-derived M1-like macrophages treated with CM (CM-LacZ, CM-CD, CM-As4257, CM-As3156) for 48 h. qPCR (**A**, **C**, and **E**) and western blot (**B**, **D**, and **F**) were used to analyze the mRNA and protein levels of M1 effector molecules. The data are presented as the mean ± standard error of three independent experiments, each performed in triplicate. (*p < 0.05 and **p < 0.01, student’s t-test)

To investigate whether CEBPD and its responsive miRNAs, miR-4257 and miR-3156, contribute to M1-like macrophage inactivation in vitro, we examined the expression of pro-inflammatory genes in M1-like macrophages cultured in conditioned medium (CM) from U87MG cells with or without CEBPD, miR-4257, or miR-3156. Higher mRNA and protein levels of pro-inflammatory effectors, including IL-12 p35, IL-12 p40, and CCR7, were detected in M1-like macrophages incubated with CM from CEBPD-deficient U87MG cells (Fig. 4A and 4B). Similar effects were observed in M1-like macrophages cultured in CM from U87MG cells lacking miR-4257 or miR-3156 (Fig. 4C–F). These results demonstrate that the pro-inflammatory effectors of M1-like macrophages are upregulated by CEBPD-, miR-4257-, or miR-3156-deficient U87MG cells in vitro.

To further support the clinical relevance of the proposed CEBPD/miR-4257/miR-3156/IL-12 regulatory axis, we examined available GBM patient datasets. In TCGA-GBM, patients with high CEBPD expression exhibit significantly poorer overall survival (Supplementary Fig. 5 A) and show markedly reduced IL12 p35 (IL12A) mRNA levels (Supplementary Fig. 5B), supporting a negative association between CEBPD and IL-12 p35 expression in human tumors. Analysis of the GSE184472 plasma dataset further demonstrates that circulating miR-4257, but not miR-3156, is significantly elevated in GBM patients compared with healthy controls (Supplementary Fig. 6), indicating that CEBPD/miR-4257/IL-12 p35 regulatory axis is clinically detectable and relevant in the GBM setting. In addition, TCGA correlation analyses show that the miR-4257 host gene ADAMTSL4 is positively associated with CEBPD and negatively associated with IL12 p35 (Supplementary Fig. 7). Together, these human datasets indirectly support our proposed regulatory axis, in which elevated CEBPD in GBM corresponds to suppressed IL-12 p35 expression, while the detectable increase of circulating miR-4257 highlights its potential involvement in GBM immunoregulatory processes.

### miR-4257 and miR-3156 are delivered by GBM-derived sEVs and induce M1 macrophage inactivation

**Fig. 5:**
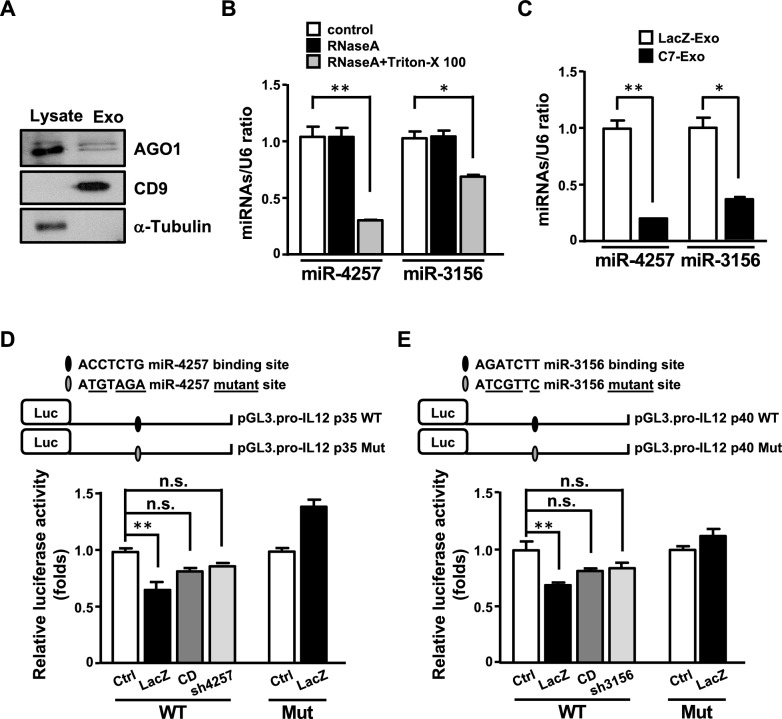
U87MG cells release sEVs containing miR-4257 and miR-3156, which target the IL-12 3’-UTR in recipient cells. (**A**) the protein expression levels of AGO1 and CD9 were detected in GBM sEVs. equal amounts of protein from whole cells and sEVs were analyzed by western blotting for AGO1 and CD9. (**B**) miR-4257 and miR-3156 were indeed present within the sEVs. sEVs were isolated from the culture medium of U87MG cells and divided into three equal portions. These samples were either untreated (control), treated with RNaseA, or treated with RNaseA plus 0.1% Triton X-100 (RNaseA Tri-X 100) for 30 min. qPCR was performed to determine the retained miRNAs in each sample. (**C**) reduced levels of miR-4257 and miR-3156 were detected in sEVs derived from U87MG cells with attenuated CEBPD expression. A comparative analysis of miR-4257 and miR-3156 levels in sEVs from U87MG cells, with or without CEBPD attenuation, was conducted. sEVs were isolated from U87MG cells harboring IPTG-inducible sh-LacZ (LacZ) or sh-CEBPD (CD) expression systems following 96 h of IPTG treatment. the miRNAs within the sEVs were extracted and quantified using qPCR. (**D**) and (**E**) U87MG-derived sEVs transport miR-4257 and miR-3156, targeting the IL-12 3’-UTR in recipient cells. sEVs were isolated from U87MG cells expressing IPTG-inducible vectors for sh-LacZ (LacZ), sh-CEBPD (CD), antisense miR-4257 (As4257), or antisense miR-3156 (As3156) after 96 h of culture in IPTG-containing medium. the luciferase activity of reporter constructs was measured following transfection with IL-12 3’UTR wild-type or mutant reporters and incubation with the sEVs for 24 h. data are presented as the mean ± SD from three independent experiments. *p < 0.05 and **p < 0.01, student’s t-test

GBM has been reported to release sEVs containing miRNAs, mRNAs, and proteins that regulate tumor progression [38]. To investigate whether GBM-secreted sEVs carry CEBPD-responsive miR-4257 and miR-3156, sEVs were isolated through sequential centrifugation. These sEVs were identified by the sEV marker protein CD9 and were found to contain Argonaute 1 (AGO1), which is required for miRNA activity (Fig. 5A). We next demonstrated that miR-4257 and miR-3156 were indeed present within the sEVs and not merely being released. Treatment of sEVs with both RNase and Triton X-100 significantly reduced miR-4257 and miR-3156 levels, whereas treatment with RNase alone caused minimal changes in miRNA levels compared to untreated sEVs (Fig. 5B). It was also found that the expression of miR-4257 and miR-3156 in sEVs from CEBPD-deficient U87MG cells was significantly decreased compared to that in sEVs from control U87MG cells (Fig. 5C). These findings demonstrate that miR-4257 and miR-3156 can be packaged into sEVs derived from GBM cells. To test whether U87MG sEVs containing miR-4257 and miR-3156 could be taken up by recipient cells, sEVs were isolated from CEBPD-, miR-4257-, or miR-3156-deficient U87MG cells. These sEVs were incubated with 293 T cells transfected with IL-12 p35 or p40 3’-UTR reporters. U87MG sEVs reduced the reporter activity of IL-12 *p35* and *p40* 3’-UTR; however, these effects were not observed in cells incubated with U87MG sEVs lacking miR-4257 and miR-3156. Meanwhile, U87MG sEVs had no effect on constructs with mutant miR-4257 or miR-3156 binding motifs (Fig. 5D and 5E). These results demonstrate that GBM sEVs deliver miR-4257 and miR-3156 into recipient cells and target the 3’-UTR of IL-12.

**Fig. 6:**
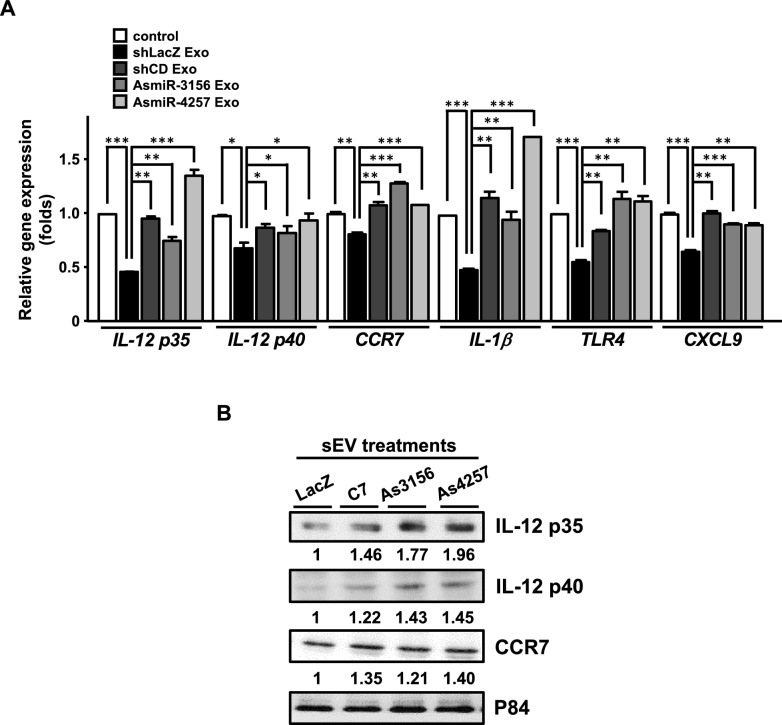
sEVs derived from CEBPD, miR-3156, or miR-4257-deficient U87MG cells induce the expression of M1 effector molecules in macrophages. (**A**) sEVs were isolated from U87MG cells expressing IPTG-inducible vectors for sh-LacZ (LacZ), sh-CEBPD (CD), antisense miR-4257 (As4257), or antisense miR-3156 (As3156) after 96 h of culture in IPTG-containing medium. total RNA was extracted from M1-like macrophages following a 48-h incubation with the sEVs. the expression levels of M1 effector molecules, including IL-12 p35, p40, CCR7, IL-1β, TLR4, and CXCL9, were analyzed using qPCR. (**B**) total protein was extracted from M1-like macrophages following a 48-h incubation with sEVs. the expression levels of M1 effector molecules, including IL-12 p35, p40, and CCR7, were analyzed by western blot. results are presented as the mean ± SD from three independent experiments. *p < 0.05, **p < 0.01, and ***p < 0.001 (Student’s t-test)

We next exposed M1-like macrophages to U87MG sEVs with or without miR-4257 and miR-3156 to determine whether these miRNAs, transported by U87MG sEVs, could alter the expression of M1-like hallmark genes. Consistently, the mRNA levels of M1-like macrophage effector molecules IL-12 p35, IL-12 p40, CCR7, IL-1β, CXCL9, and TLR4 were suppressed by U87MG sEVs, but not by sEVs from CEBPD-, miR-4257-, or miR-3156-deficient U87MG cells (Fig. 6A). The higher protein levels of IL-12 p35, IL-12 p40, and CCR7 were observed in M1-like macrophage incubated with sEVs from CEBPD, miR-4257 or miR-3156 deficient U87MG cells compared to control U87MG sEVs (Fig. 6B). Collectively, our findings indicate that GBM cells release sEVs containing CEBPD-responsive miR-4257 and miR-3156, which functionally inactivate M1-like macrophages by repressing IL-12.

### Inhibiting miR-4257 or miR-3156 in GBM cells activates tumor-suppressive M1-like macrophages in vivo

**Fig. 7:**
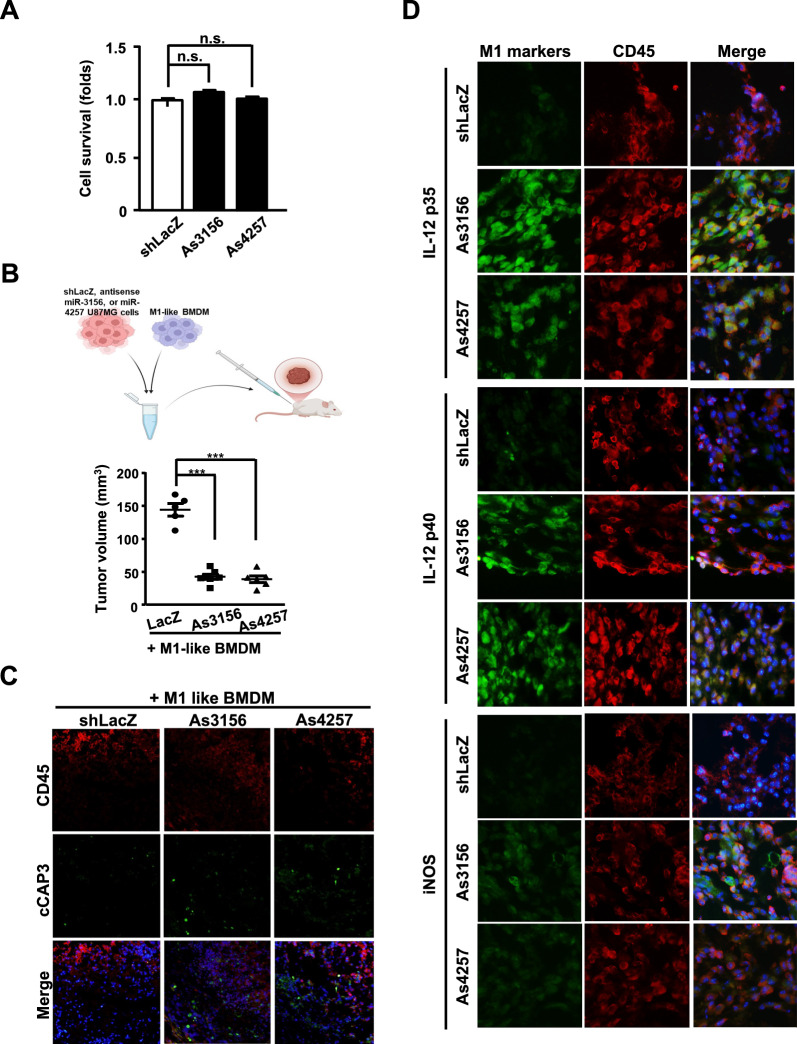
Inhibition of miR-4257 or miR-3156 in U87MG cells activates M1-like macrophages and suppresses tumor growth in vivo. Inhibition of miR-4257 or miR-3156 in U87MG cells does not affect cell viability. (**A**) CCK-8 assay was performed to evaluate cell survival. (**B**) blocking miR-4257 or miR-3156 in U87MG cells reduces tumor volume in vivo. U87MG cells harboring IPTG-inducible vectors for sh-LacZ (LacZ), antisense miR-4257 (As4257), or antisense miR-3156 (As3156) were cultured in IPTG-containing medium for 96 h. These cells were subcutaneously co-inoculated with M1-like bone marrow-derived macrophages into NOD-SCID mice. A schematic illustration of the animal model experiment for glioma, demonstrating that the inactivation of miR-3156 and miR-4257 suppresses M1-like macrophage activation. on day 14, tumor size was measured using external calipers, and tumor volume was calculated using the standard formula: V = (w × l^2^) × 0.52, where l is the length and w is the width of the tumor (n = 5–6 per group). (**C**) histological analysis of tumor cell apoptosis in xenografts derived from sh-LacZ-, As3156-, and As4257-U87MG cells co-inoculated with M1-like macrophages was performed. Tumor tissues were subjected to immunofluorescence staining using antibodies specific for cleaved caspase-3 (cCap3; active caspase-3) and CD45 (a marker for xenograft M1 macrophages). DAPI staining was used to visualize cell nuclei. (**D**) loss of miR-3156 or miR-4257 enhances the expression of IL-12 p35, p40, and iNOS, which colocalize with CD45. ***p < 0.001 (Student’s t-test)

As GBM cells can release sEVs containing miR-4257 and miR-3156 to inactivate M1 macrophages in vitro, we further examined whether this effect could be detected in vivo. At the beginning of this experiment, we assessed whether miR-4257 and miR-3156 antisense (As4257 and As3156) could affect cell survival in U87MG cells. We found that As4257 and As3156 did not affect cell viability (Fig. 7A). As expected, reduced xenograft tumor volume was observed in miR-4257- or miR-3156-deficient U87MG cells co-inoculated with M1-like macrophages, compared to the control group (Fig. 7B). Moreover, caspase-3 activity was increased in tumor sections of miR-4257- or miR-3156-deficient U87MG cells (Fig. 7C). To further validate whether miR-4257 and miR-3156 in GBM inactivate tumor-inhibitory M1-like macrophages, we used an immunofluorescence assay to stain hallmark genes of M1-like macrophages in tumor histology. Importantly, higher levels of IL-12 p35, IL-12 p40, and iNOS were detected in the macrophages co-inoculated with miR-4257- or miR-3156-deficient U87MG cells in vivo (Fig. 7D). Collectively, these results suggest that miR-4257 and miR-3156 in GBM suppress the tumor-inhibitory effect and hallmark effector molecules of M1-like macrophages in vivo.

## Discussion

Glioblastoma is a highly heterogeneous tumor that profoundly influences the immune response and drives tumor progression [5, 38]. Notably, tumor-associated macrophages (TAMs) are classified into two phenotypic subtypes: M1 macrophages, which exhibit anti-tumor properties, and M2 macrophages, which promote tumor progression [39]. The balance between these subtypes significantly determines tumor progression. MicroRNAs (miRNAs) play critical roles in cellular physiology by regulating gene expression at the transcriptional and post-transcriptional levels. Intriguingly, previous studies have demonstrated that miRNAs can be secreted by glioma cells via sEV transmission, facilitating tumor progression [40–42].

CEBPD, a transcription factor, is upregulated in glioma cells and has been implicated in tumor progression, including cancer stemness and resistance to chemotherapy [2, 43]. Additionally, CEBPD modulates microRNA transcription to influence various cellular processes. For instance, it regulates the transcription of miR-744, miR-3154, and miR-3162 to mediate epigenetic regulation, thereby promoting bortezomib-induced leukemic cell arrest and cell death [25]. CEBPD also regulates miR-135a to promote angiogenesis and reduce axon outgrowth in an Alzheimer’s disease model [33]. In this study, we demonstrate that glioma-derived CEBPD regulates the transcription of miR-4257 and miR-3156. These miRNAs are subsequently secreted via sEVs and bind to *IL-12* 3’-UTR in macrophages, resulting in immunosuppressive effects. Previously, GC binding protein (GC-BP), a novel zinc finger nuclear factor, was shown to selectively inhibit *IL-12 p35* gene transcription by binding to its promoter in BMDMs [44]. Similarly, the human erythroid Kruppel-like factor (EKLF) was found to inhibit the transcriptional activity of the *IL-12 p40* promoter in mouse macrophages [45]. Despite these findings, our study reveals a novel "outside-in" mechanism of post-transcriptional regulation of IL-12 expression in macrophages.

Extracellular vesicles include sEVs, microvesicles, and apoptotic bodies [42]. Among these, sEVs are particularly notable for containing microRNAs, mRNAs, and proteins [42], which can be delivered to recipient cells, thereby facilitating intercellular communication. In glioma, sEV microRNAs have been shown to promote tumor proliferation, migration, invasion, and immunosuppression [46–48]. These sEVs can be secreted by glioma cells or tumor-associated macrophages (TAMs) [46]. However, further research is required to elucidate the underlying mechanisms and phenotypic interactions among glioma, sEV microRNAs, and immunosuppression. Our results demonstrated that miR-4257 and miR-3156 bind to the 3’-UTR of *IL-12* in macrophages, thereby suppressing M1 macrophage phenotypes. These microRNAs are located within the genes *ADAMTSL4* and *ANKRD30BP3*, respectively. Furthermore, miR-21 has been reported to inhibit *IL-12* transcription in allergic airway inflammation [49]. Notably, miR-21 is upregulated in glioma, where it reduces cell death and promotes cell migration [50]. In this study, our findings uncover a pioneering mechanism in which miR-4257 and miR-3156 regulate *IL-12* transcription in glioma.

Immunosuppression presents a major challenge in glioblastoma treatment, including the accumulation of tumor-associated myeloid cells (TAMCs), which comprise tumor-associated macrophages (TAMs) and microglia [51]. To counteract the immunosuppressive environment and enhance therapeutic efficacy, IL-12—a potent tumor-suppressive cytokine secreted by macrophages that directly sustains persistent cytotoxic T-cell activity [36] has been used to treat glioma patients [36, 51, 52]. Previously, the combination of intratumoral IL-12 cytokine and αPD-1 inhibitors was shown to enhance anti-tumor immunity in mouse glioma models [53]. Additionally, IL-12 has been found to stimulate IFN-γ secretion by natural killer (NK) cells and Th1 cells, thereby activating phagocytes to control parasite growth [54]. In our study, we discovered that IL-12 levels can be suppressed by CEBPD-upregulated miR-4257 and miR-3156 binding to *IL-12* 3’-UTR in glioma. Following the uptake of miR-4257 and miR-3156, the inactivation of IL-12 in M1 macrophages shows an M2-like M1 macrophage phenotype. It supports that CEBPD plays a protumor role in the tumor microenvironment. Furthermore, the development of oligonucleotide-based medicines is an emerging therapeutic strategy for various diseases, including neurodegenerative and infectious diseases [55, 56]. Recent studies have demonstrated that antisense oligonucleotides can be incorporated into nanoparticles or engineered sEVs to enhance their delivery across the blood–brain barrier and improve stability and cellular uptake [57, 58]. These nanocarrier or engineered exosome-based strategies hold promise for achieving efficient and targeted delivery of ASO therapeutics to the central nervous system, thereby increasing their translational potential for treating glioma. In this context, antisense oligonucleotides targeting miR-4257 and miR-3156 could be developed as a potential treatment for glioma, enhancing glioma immunotherapy.

**Fig. 8:**
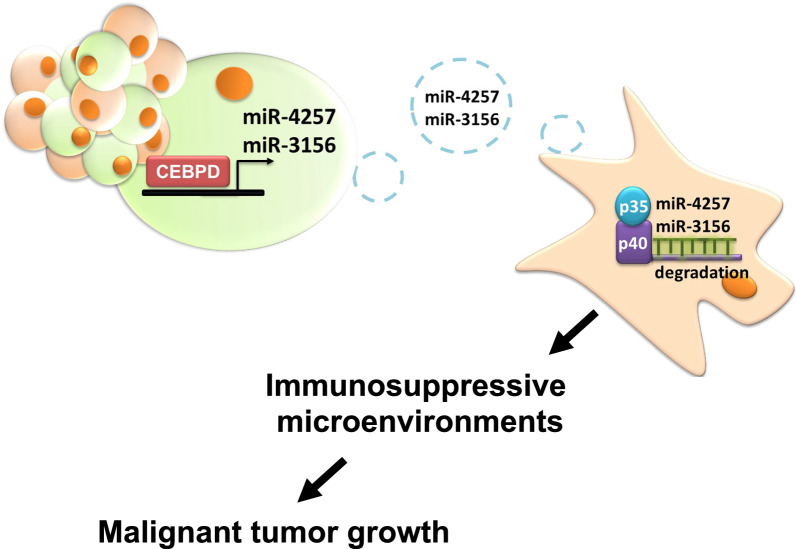
Schematic diagram illustrating the effect of glioma CEBPD suppression on immune response through sEV miR-4257/miR-3156 targeting of IL-12 in M1 macrophages. Glioma CEBPD activates the transcription of miR-4257 and miR-3156 through direct regulation. These microRNAs, transmitted via sEVs, target M1 macrophages to reduce IL-12 transcription and expression. This pathway demonstrates that glioma CEBPD contributes to immunosuppression by regulating microRNAs and targeting tumor-associated macrophages through sEV transmission

In conclusion, this study is the first to elucidate the CEBPD-involved immunosuppressive effects in glioma. We demonstrate that CEBPD directly regulates the transcription of miR-4257 and miR-3156 in glioma cells, which can be delivered via sEVs to target the 3’-UTR of IL-12 in macrophages. Knockdown of CEBPD in glioma reduces M1 macrophage activity and inhibits tumor formation (Fig. 8). These findings provide novel insights into the CEBPD/miRs-4257 and −3156/IL-12 pathways and their potential role in glioma immunotherapy.

## Supplementary Information

Additional file 1

Additional file 2

Additional file 3

## Data Availability

The data supporting this study are available upon reasonable request to the corresponding author.
