# Supplementary figures and images for: CCAAT/enhancer-binding protein delta regulates miRs-4257 and 3156 to attenuate the interleukin 12 through small extracellular vesicle transmission in glioblastoma
Source: Cancer Cell Int. 2026 Feb 25;26:149. doi: 10.1186/s12935-026-04225-2 (PMC13041471; doi:10.1186/s12935-026-04225-2)

Figure 4B

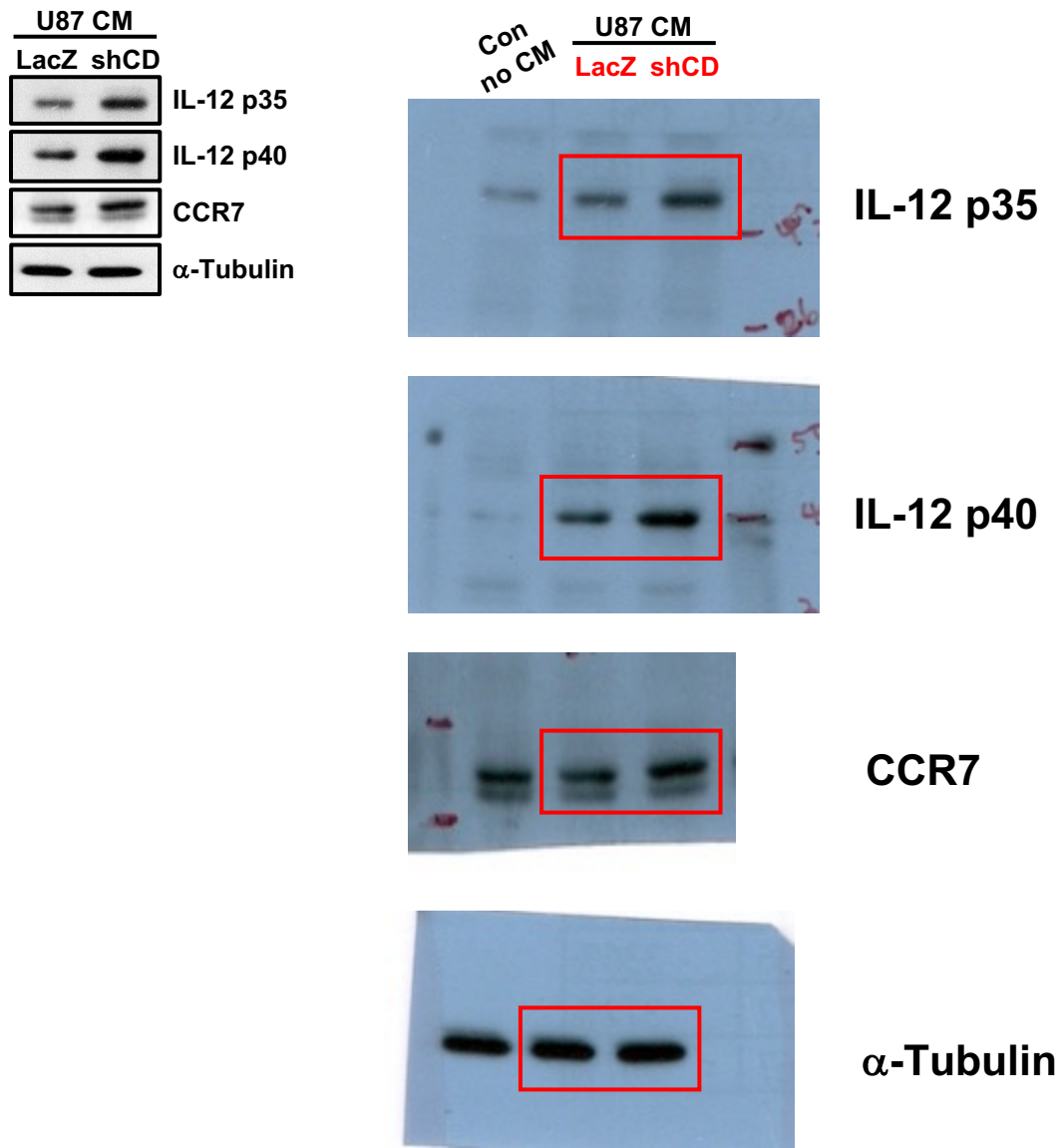

Figure 4D

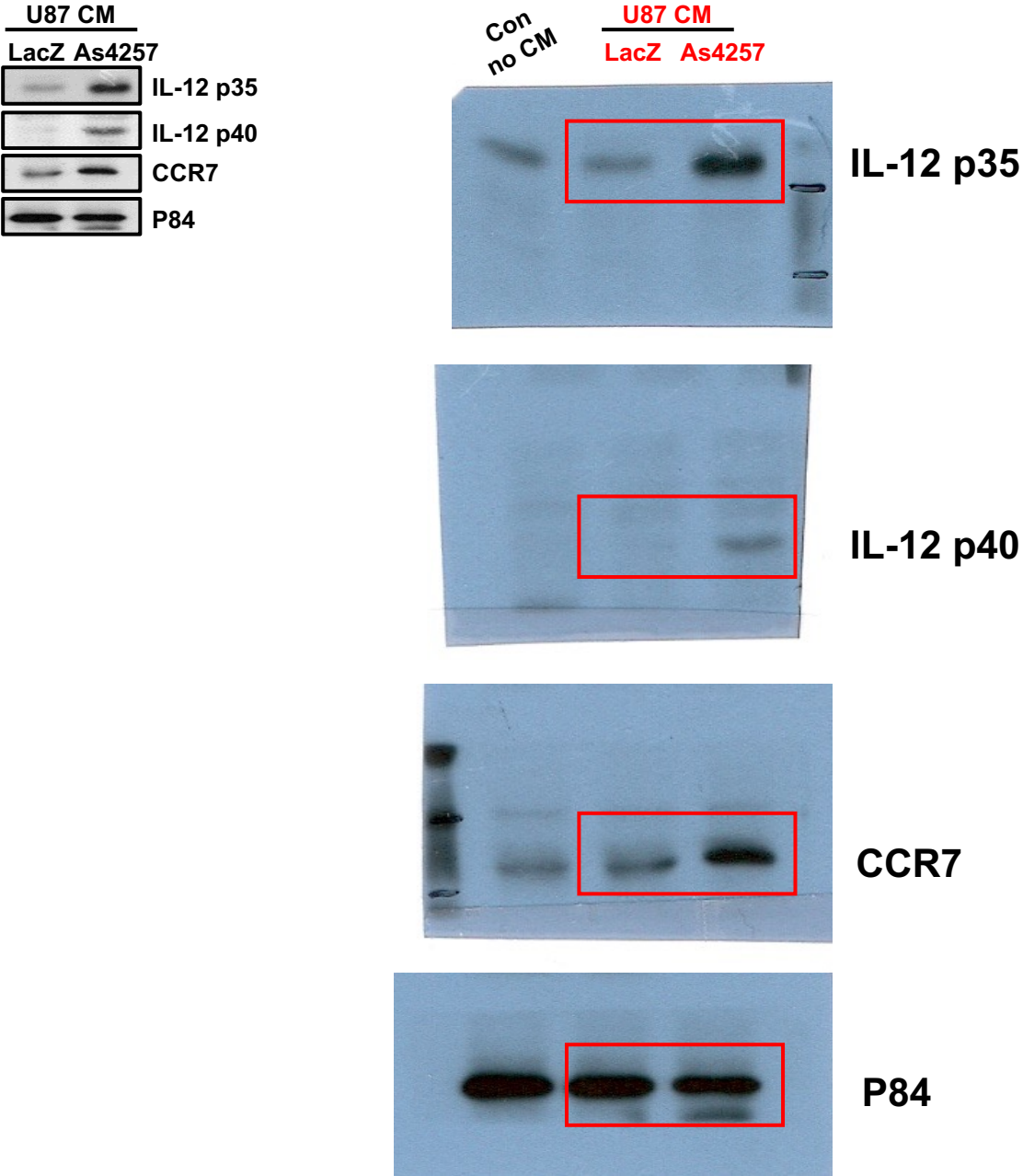

Figure 4F

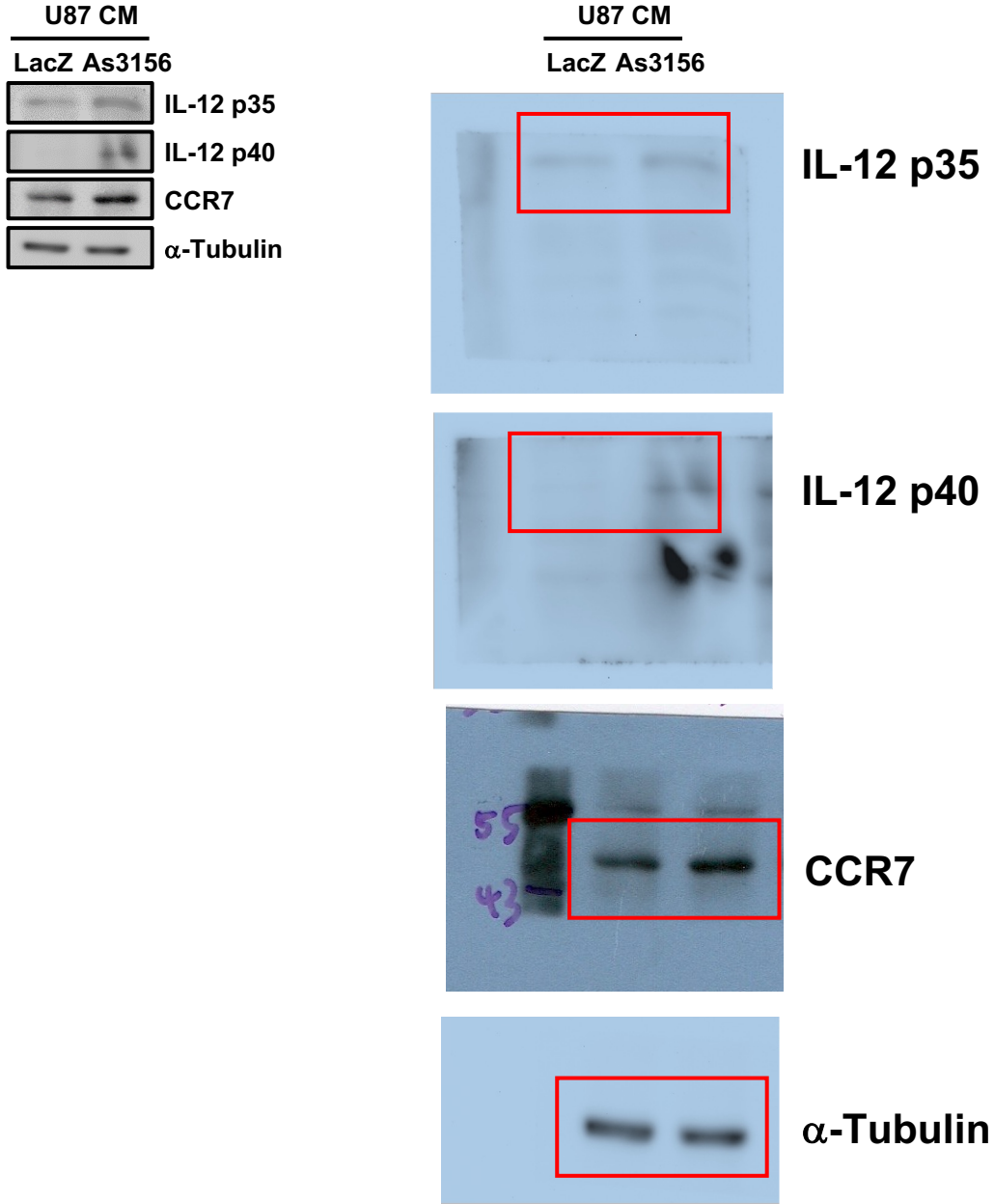

**Fig 5A**

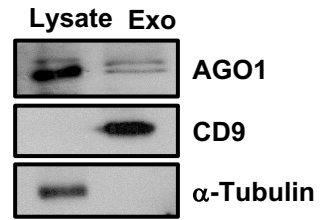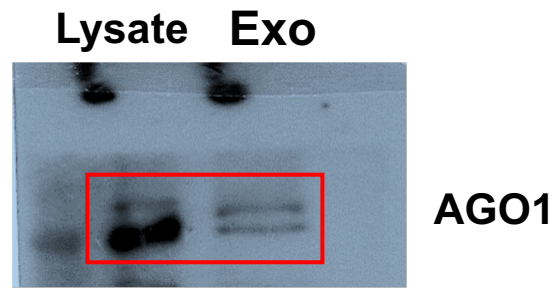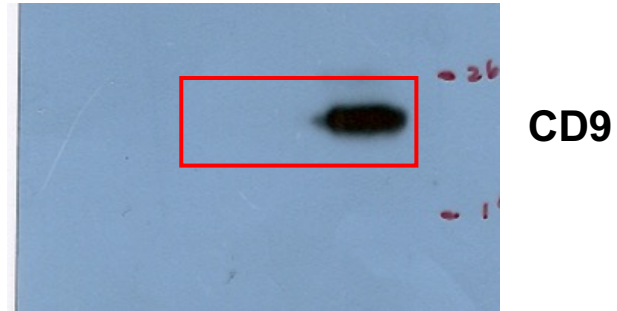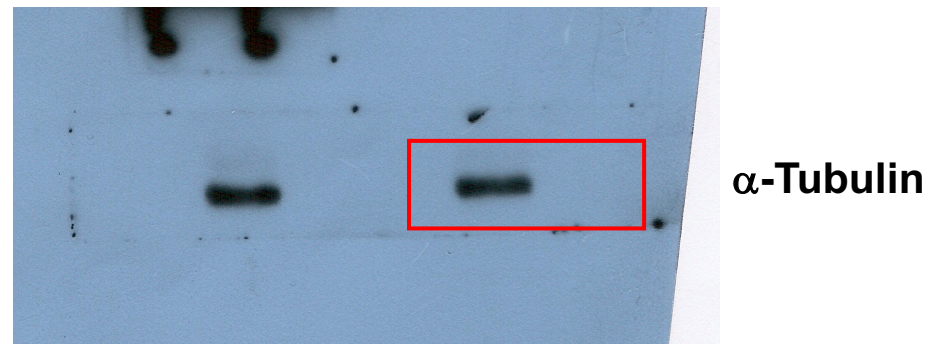

Fig 6B

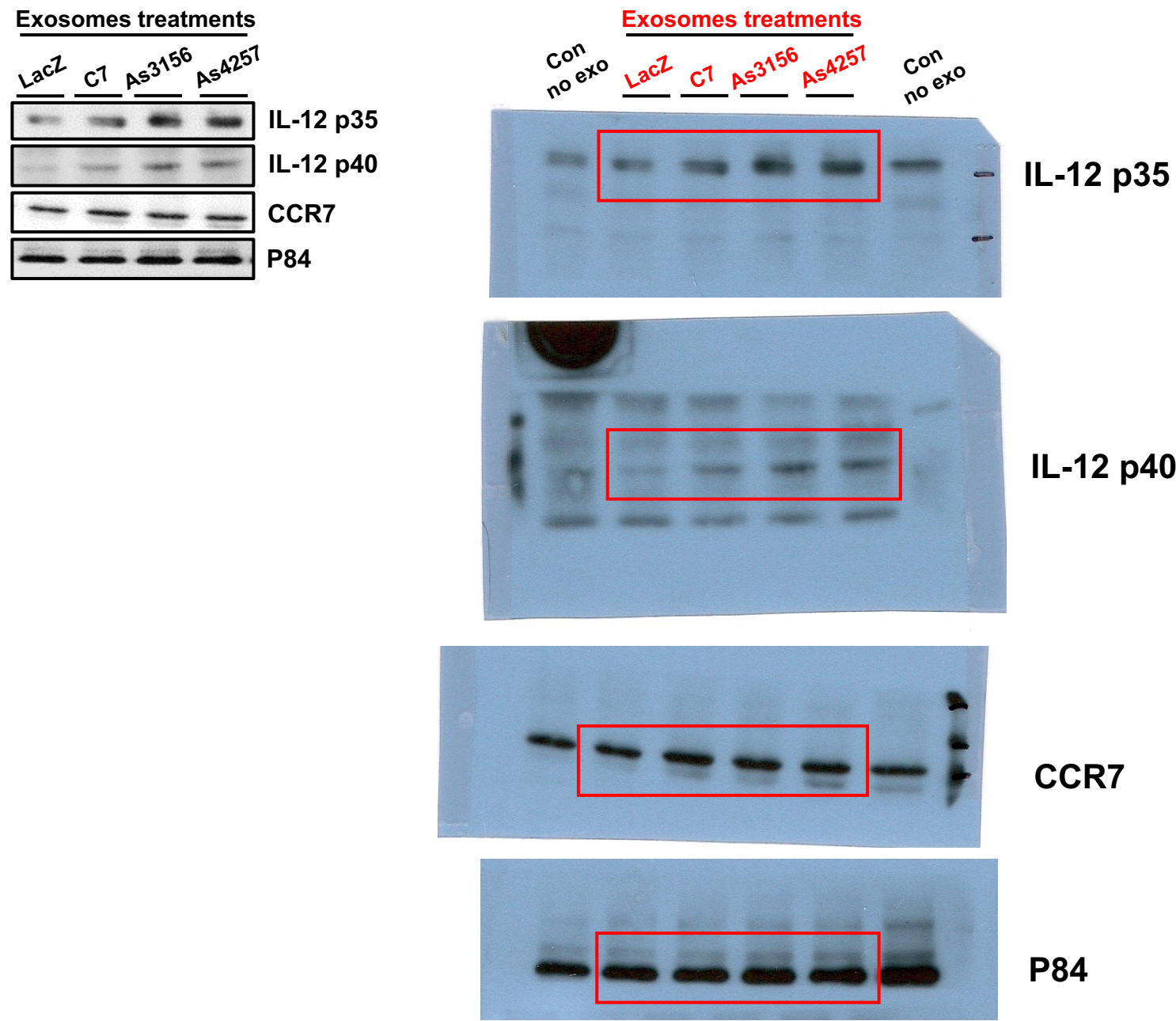

**Figure 3A**

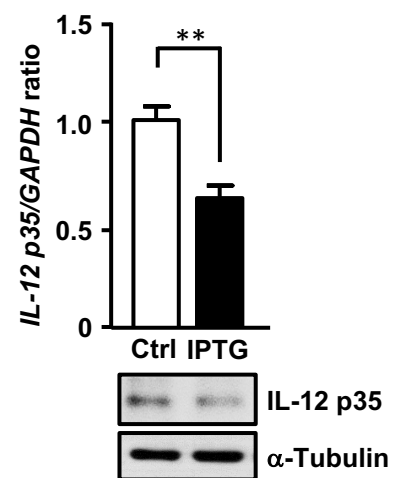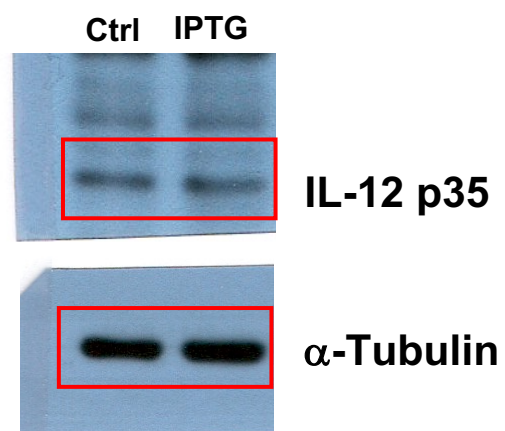

**Figure 3B**

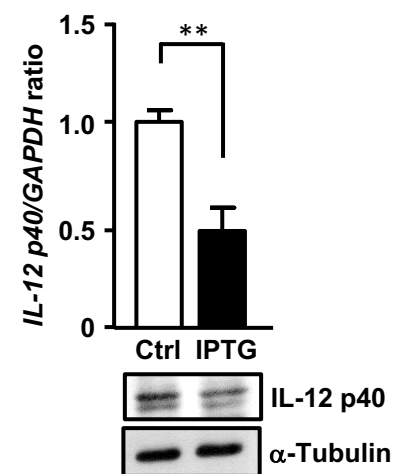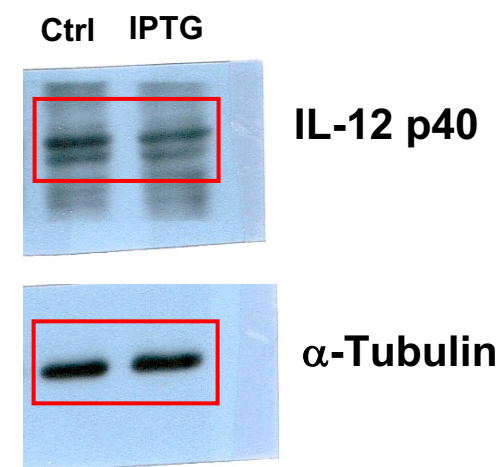

Supplement: Supplementary file 1 — Additional file 1 [file 12935_2026_4225_MOESM1_ESM.pdf]
