# Supplementary material for: CCAAT/enhancer-binding protein delta regulates miRs-4257 and 3156 to attenuate the interleukin 12 through small extracellular vesicle transmission in glioblastoma
Source: Cancer Cell Int. 2026 Feb 25;26:149. doi: 10.1186/s12935-026-04225-2 (PMC13041471; doi:10.1186/s12935-026-04225-2)
Supplement: Supplementary file 2 — Additional file 2 [file 12935_2026_4225_MOESM2_ESM.pdf]

Figure 1A

		L	W	volume
shLacz+M1 like BMDM	**1**	6.5	5.8	109.33
	**2**	6.3	5.4	91.854
	**3**	6.5	5.7	105.5925
	**4**	5.6	5.5	84.7
		**L**	**W**	**volume**
shCD+M1 like BMDM	**5**	5	3.9	38.025
	**6**	4.5	4.3	41.6025
	**7**	5.7	4.7	62.9565
	**8**	5.7	4.5	57.7125

Figure 7B

		L	W	volume
Lacz+M1 like BMDM	**1**	6.8	6.6	148.104
	**2**	7	6.9	166.635
	**3**	6.8	6.8	157.216
	**4**	7	6.2	134.54
	**5**	6.7	5.8	112.694
		L	W	volume
AS3156+M1 like BMDM	**6**	5	4.5	50.625
	**7**	5.4	4.7	59.643
	**8**	4.7	4.2	41.454
	**9**	4.6	4.2	40.572
	**10**	4.9	3.3	26.6805
	**11**	4.8	4.2	42.336
	**12**	4.9	4.2	43.218
		L	W	volume
AS4257+M1 like BMDM	**13**	5.7	3.4	32.946
	**14**	5.1	3.9	38.7855
	**15**	3.6	3.6	23.328
	**16**	4.9	4.2	43.218
	**17**	4.9	4.9	58.8245
	**18**	5	4.1	42.025
