# Supplementary material for: CCAAT/enhancer-binding protein delta regulates miRs-4257 and 3156 to attenuate the interleukin 12 through small extracellular vesicle transmission in glioblastoma
Source: Cancer Cell Int. 2026 Feb 25;26:149. doi: 10.1186/s12935-026-04225-2 (PMC13041471; doi:10.1186/s12935-026-04225-2)
Supplement: Supplementary file 3 — Additional file 3 [file 12935_2026_4225_MOESM3_ESM.pdf]

Supplementary table. 1 Up-regulated miRNAs by CEBPD in U373MG cells but not in THP-1 cells.

Name of miRNAs	Fold change in U373MG cells	Fold change in THP-1 cells
hsa-miR-1193	1.7	0.69
hsa-miR-1254	1.6	undetectable
hsa-miR-1321	1.71	0.75
hsa-miR-135a	1.6	0.78
hsa-miR-184	1.43	undetectable
hsa-miR-198	1.74	0.91
hsa-miR-2116	1.71	undetectable
hsa-miR-3125	1.54	1.03
hsa-miR-3126	1.47	1.02
hsa-miR-3144	1.55	0.83
hsa-miR-3156	1.49	0.95
hsa-miR-3176	1.44	0.93
hsa-miR-3178	1.46	0.62
hsa-miR-3190	1.62	1.02
hsa-miR-3192	1.43	undetectable
hsa-miR-323	1.35	0.8
hsa-miR-4257	1.51	1.05
hsa-miR-4294	1.47	0.95
hsa-miR-4299	1.69	0.97
hsa-miR-4300	1.61	0.99
hsa-miR-486	1.33	undetectable
hsa-miR-505	1.42	0.87
hsa-miR-514b	1.59	1.18
hsa-miR-877	1.89	1.03
hsa-miR-885	1.24	0.95
hsa-miR-921	1.57	0.93

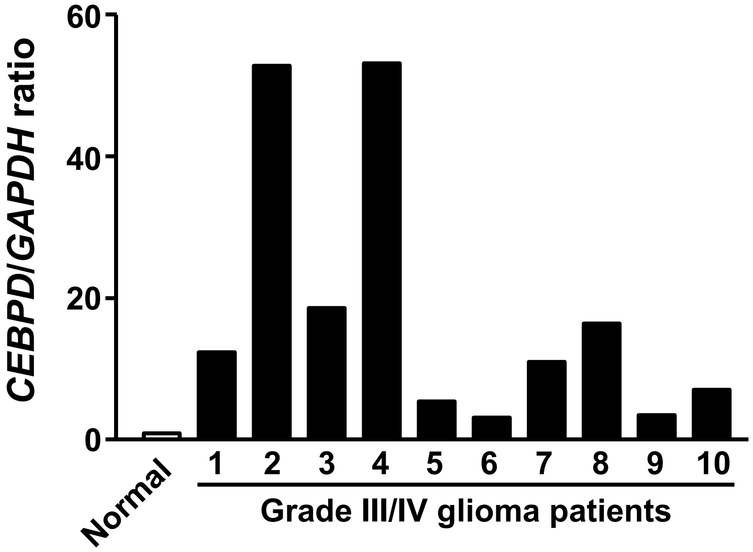

Supplementary Figure 1 The expression of CEBPD is increased in GBM patients relative to normal tissues. The mRNA level of CEBPD is elevated in GBM patients compared to normal brain tissue. qPCR analysis was performed to examine the mRNA level of CEBPD in both GBM and normal tissues.

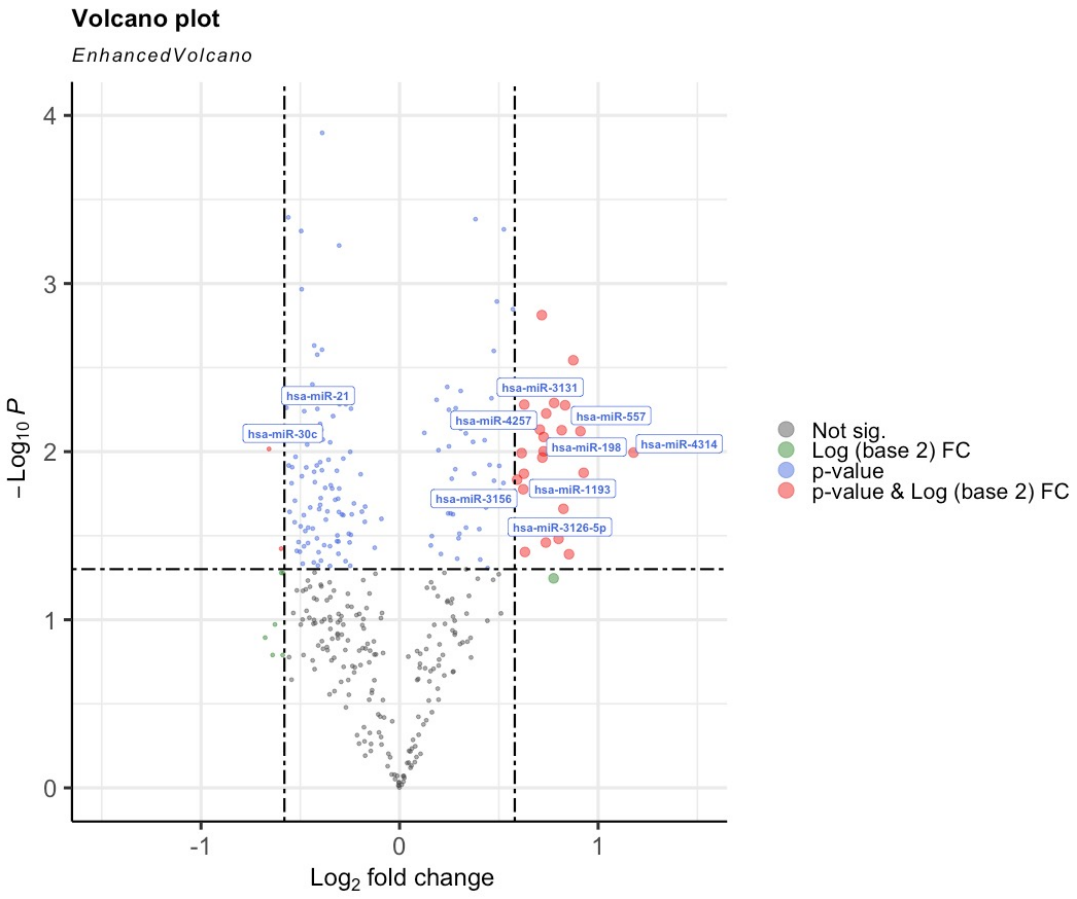

Supplementary Figure 2 Volcano plot showing differential expression of miRNAs regulated by CEBPD in U373MG cells.

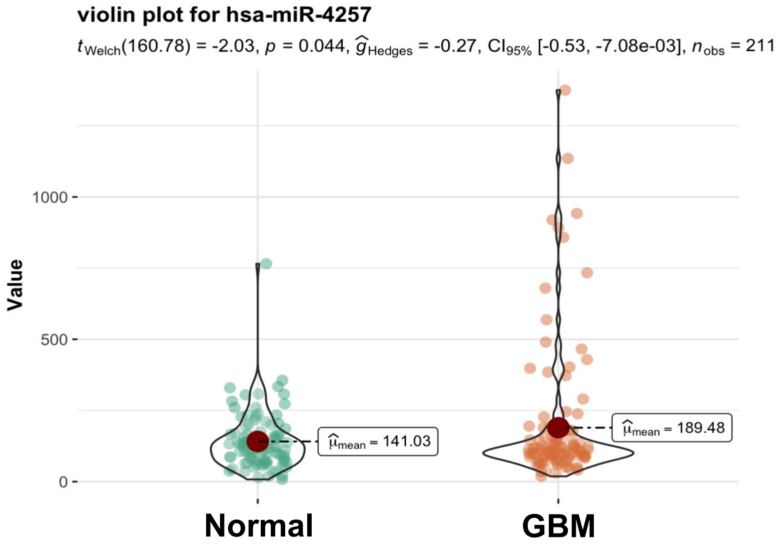

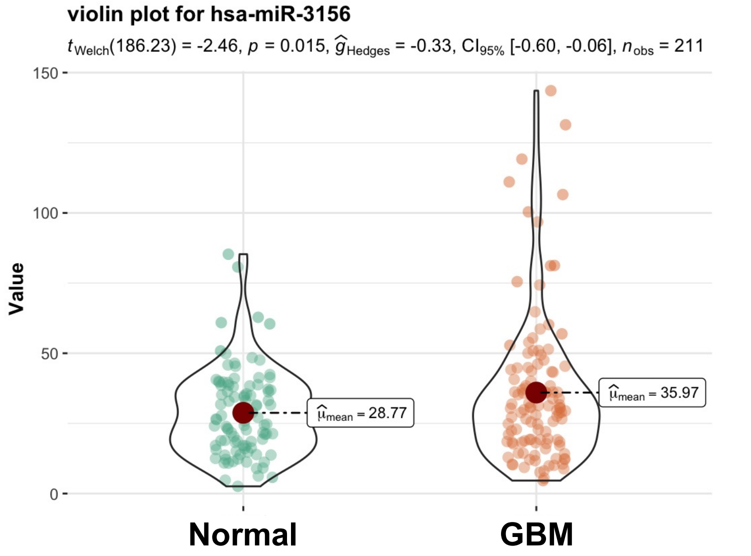

Supplementary Figure 3. Violin plot of miRNA expression between control and GBM groups in the GSE145510 dataset. Differential expression of hsa-miR-4257 and hsa-miR-3156 between the two groups is shown, respectively. (Student's t-test).

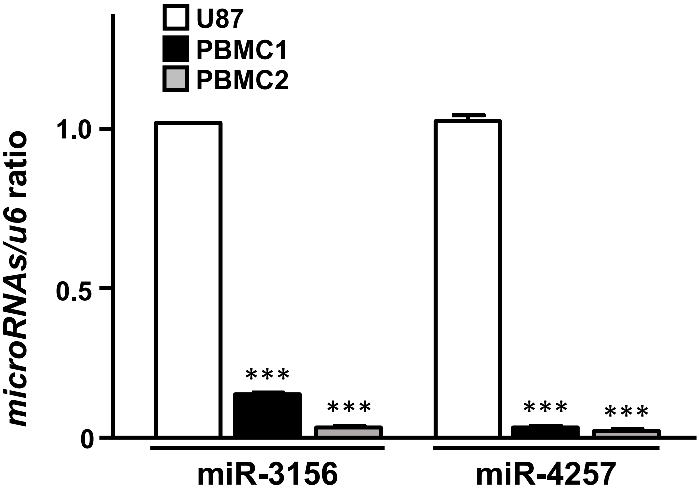

Supplementary Figure 4. Preferential expression of miR-4257 and miR-3156 in glioblastoma cells. qPCR was used to analyze the levels of miR-4257 and miR-3156 in U87MG cells and human peripheral blood monocytes. Results are presented as means ± SEM of three independent experiments (**p* < 0.05, ***p* < 0.01, ***p < 0.001, Student's t-test).

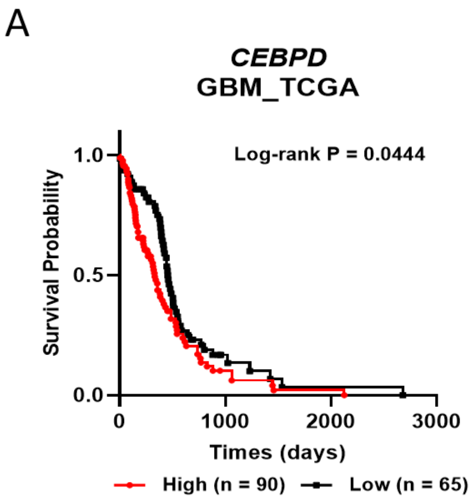

Supplementary Figure 5 In TCGA-GBM, higher CEBPD expression is associated with significantly poorer overall survival and correlates with lower IL12A expression. (A) TCGA-GBM Overall Survival by CEBPD Expression. The overall survival data of GBM patients from the TCGA cohort (accessed via https://tcga-survival.com). Patients were stratified into CEBPD-high (n=90) and CEBPD-low (n=65) groups using the optimal cutoff. The Kaplan–Meier plot demonstrates a noticeable separation between the two curves, with the CEBPD-high group exhibiting shorter survival durations. (B) CEBPD Expression Negatively Correlates with IL12A in TCGA-GBM. IL12A mRNA expression levels in 166 TCGA-GBM samples (retrieved from FireBrowse). Patients were divided into CEBPD-high (n=83) and CEBPD-low (n=83) groups using the median CEBPD expression as cutoff. IL12A levels were markedly reduced in the CEBPD-high group.

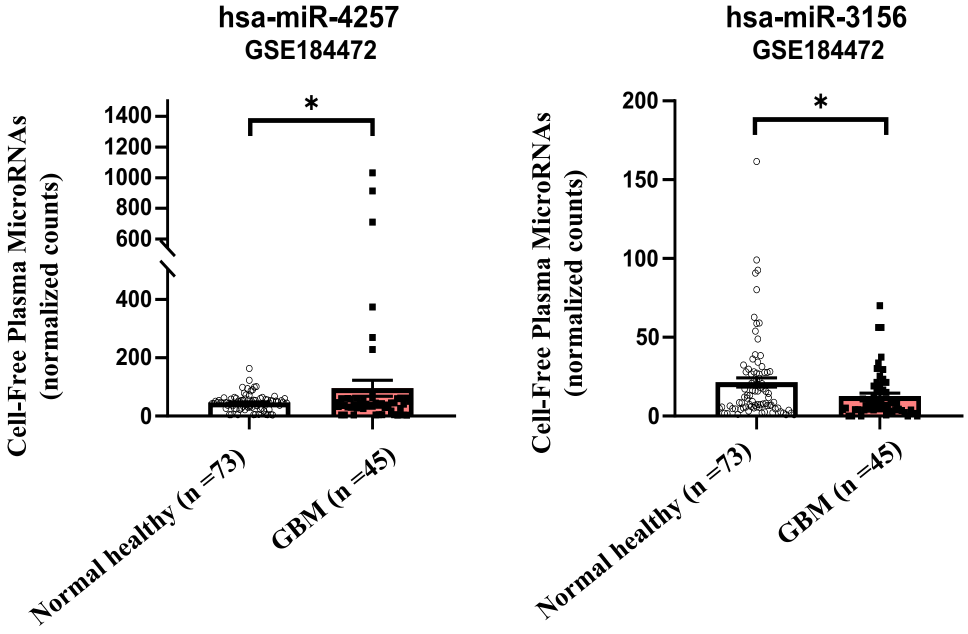

Supplementary Figure 6 The plasma miR-4257 and miR-3156 expression in GBM plasma samples. From the GSE184472 dataset, the comparison between healthy controls and GBM patients shows that hsa-miR-4257, but not miR-3156, is significantly elevated in GBM plasma samples.

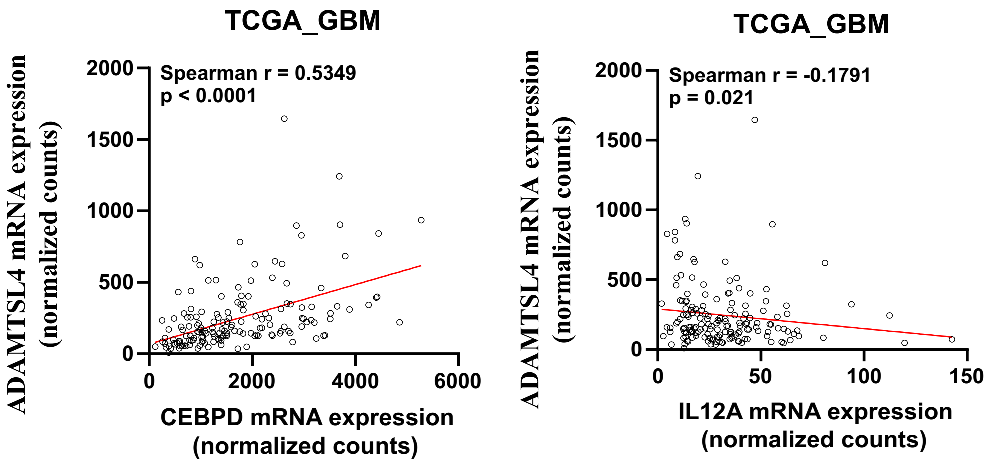

Supplementary Figure 7 Clinical relevance of ADAMTSL4, CEBPD, and IL-12A in GBM. Correlation analysis in the TCGA-GBM cohort demonstrates that ADAMTSL4 (the host gene of miR-4257) is positively associated with CEBPD expression and negatively associated with IL12A expression, supporting the proposed CEBPD/miR-4257/IL-12A regulatory axis in GBM.
